# Variation in Foot Strike Patterns among Habitually Barefoot and Shod Runners in Kenya

**DOI:** 10.1371/journal.pone.0131354

**Published:** 2015-07-08

**Authors:** Daniel E. Lieberman, Eric R. Castillo, Erik Otarola-Castillo, Meshack K. Sang, Timothy K. Sigei, Robert Ojiambo, Paul Okutoyi, Yannis Pitsiladis

**Affiliations:** 1 Department of Human Evolutionary Biology, Harvard University, Cambridge, Massachusetts, United States of America; 2 Medical Physiology Department, School of Medicine, Moi University, Eldoret, Kenya; 3 Department of Statistics and Computer Science Moi University, Eldoret, Kenya; 4 Department of Orthopaedics and Rehabilitation, School of Medicine, Moi University, Eldoret, Kenya; 5 Centre for Sport and Exercise Science and Medicine, University of Brighton, Brighton, United Kingdom; University of California, Merced, UNITED STATES

## Abstract

Runners are often categorized as forefoot, midfoot or rearfoot strikers, but how much and why do individuals vary in foot strike patterns when running on level terrain? This study used general linear mixed-effects models to explore both intra- and inter-individual variations in foot strike pattern among 48 Kalenjin-speaking participants from Kenya who varied in age, sex, body mass, height, running history, and habitual use of footwear. High speed video was used to measure lower extremity kinematics at ground contact in the sagittal plane while participants ran down 13 meter-long tracks with three variables independently controlled: speed, track stiffness, and step frequency. 72% of the habitually barefoot and 32% of the habitually shod participants used multiple strike types, with significantly higher levels of foot strike variation among individuals who ran less frequently and who used lower step frequencies. There was no effect of sex, age, height or weight on foot strike angle, but individuals were more likely to midfoot or forefoot strike when they ran on a stiff surface, had a high preferred stride frequency, were habitually barefoot, and had more experience running. It is hypothesized that strike type variation during running, including a more frequent use of forefoot and midfoot strikes, used to be greater before the introduction of cushioned shoes and paved surfaces.

## Introduction

Runners are commonly categorized according to strike type (also known as footfall pattern), and it is widely observed that more than 85% of habitually shod runners typically rearfoot strike (RFS), in which the heel is the first part of the foot to contact the ground [1,2]. In contrast, some runners (many of them elite athletes) have been observed to forefoot strike (FFS), in which the ball of the foot lands before the heel, or to midfoot strike (MFS), in which the heel and ball of the foot land almost simultaneously [3]. In addition, numerous studies have found that barefoot and minimally shod runners are more likely than habitually shod runners to MFS or FFS [4–13]. However, some habitually barefoot individuals have been observed to primarily RFS when they run [14], and people in minimal shoes are more likely to run with a RFS than those who are barefoot [15].

Differences in strike patterns have led to numerous hypotheses about their relative costs and benefits. Although FFS and RFS landings do not differ in terms of economy [16–19], FFS and some MFS landings differ from RFS landings in generating no discernible impact peak in the vertical ground reaction force just after contact. Whether the rate of loading and magnitude of impact peaks contribute to repetitive stress injuries is debated [20–23], but impact peaks can be uncomfortable, often causing barefoot runners to avoid RFS landings on hard surfaces without a cushioned shoe [7–10, 20–23].

Regardless of the advantages and disadvantages of FFS, MFS and RFS landings, one issue that has been insufficiently considered is variation, both within and between individuals. How much do runners vary their strike patterns, and what causes this variation? Although runners tend to be characterized as either rearfoot, midfoot or forefoot strikers, it is likely that most use all three kinds of strikes but in different proportions and contexts. All people FFS when running up a steep incline, and the tendency to RFS is often greater when descending [24,25]. In addition, runners are more likely to MFS or FFS as they increase speed [26]. Additional factors that may affect strike type include training and skill, fatigue, the presence of shoes, shoe design, and substrate characteristics such as stiffness, slipperiness, unevenness and roughness. For example, habitually shod people who normally RFS typically switch to a FFS when asked to run barefoot on hard surfaces such as asphalt, but often continue to RFS when running barefoot on less stiff surfaces such as grass or cushioned mats [7–9]. Evidence that minimally shod individuals are more than twice as likely to RFS as barefoot individuals [7–9,15] suggests that sensory feedback from the foot strongly influences strike type.

The goal of this study therefore was to explore how much runners vary strike type on level surfaces, and to test some of the factors that may contribute to this variation. Conceptually, the factors that influence strike type variation can be classified into three non-mutually exclusive categories: *intrinsic*, *extrinsic* and *acquired*. Intrinsic factors relevant to strike type are characteristics of the runner that are not under control such as height, sex, age, and body mass. The dominant extrinsic factors relevant to strike type are characteristics of a runner’s environment that potentially affect kinematics such as the nature of the substrate (e.g., surface stiffness, slope, unevenness, slipperiness) as well as footwear characteristics such as heel cushioning that may affect how the body interacts with the ground. Speed can also be an extrinsic factor depending on circumstance (e.g., when a runner is required to run faster or slower, as in this experiment). Finally, acquired factors are characteristics that a runner develops or learns. Some acquired characteristics, such as running history, footwear history, physical fitness, strength, and existing injuries, are often a product of an individual’s background. Others such as step frequency may be modifiable characteristics—skills—that are acquired through cultural processes such as coaching, imitation, practice, and experimentation [27].

Using this conceptual framework, we tested two general hypotheses about intra- and inter-individual strike type variation among a diverse sample of individuals who varied in several intrinsic, extrinsic and acquired characteristics, and for whom we experimentally modified several extrinsic and acquired variables including surface stiffness, speed, and step frequency. The first general hypothesis (H1) is that extrinsic, intrinsic, and acquired factors influence the degree of intra-individual variation in strike type. Specifically, because shoes slow the rate of impact loading, limit exteroreception, and mitigate the effects of substrate variations on the foot and the rest of the body, H1a predicts that individuals who are barefoot will use more varied foot strike patterns than individuals who are shod. In addition, because speed and surface stiffness may affect aspects of kinematics and kinetics relevant to strike [7–9, 26], H1b predicts that runners are likely to use more varied strike patterns on soft surfaces and at slower speeds.

The second general hypothesis (H2) is that a combination of extrinsic, intrinsic, and acquired factors are predictive of foot strike angle as well as strike type both within and between individuals. Specifically, we predict that runners are more likely to FFS as they increase speed, increase step frequency, and run on harder surfaces. In addition, because impact peaks can cause discomfort and might contribute to overuse injuries, especially in unshod individuals, we predict that runners who are habitually barefoot and run more regularly over longer distances are more likely to FFS independent of other intrinsic factors such as sex, age, and body shape and size variation.

## Materials and Methods

### Study Design

Although kinematic variables such as foot strike are often compared between groups that differ in terms of footwear use (e.g., [4–8,11,12,15]), the hypotheses this study tests require a combined within- and between- subjects experimental design. In particular, we asked subjects who varied in terms of the intrinsic and acquired factors described above to perform a set of trials that independently varied three external factors: speed, surface stiffness and step frequency. Although this study design requires repeated measurements, which can be accounted for statistically using General Linear Mixed Models [36], it avoids potential sampling problems, such as heterogeneity within and between groups as well as assignment bias.

### Participants

Because this study explores both intra- and inter-individual variation, it is necessary to test the above hypotheses with an appropriate population that varies considerably in a range of extrinsic, intrinsic, and acquired factors including footwear use. Almost all people in developed nations are habitually shod, and although barefoot running has recently gained popularity in countries such as the US, few if any of these barefoot enthusiasts grew up unshod, and some may have consciously adopted a running form advocated by books or websites. At the other end of the continuum, most habitually barefoot populations do not practice much long distance running. For this reason, we chose to focus on Kalenjin-speaking communities from Western part of Kenya, an area of special relevance for the questions posed by this study because of the considerable variation in footwear usage and running habits within this population, which includes many of the world’s best distance runners, most of whom grew up barefoot [27,28].

48 Kalenjin individuals (Table 1) were recruited from the region around Eldoret in the Uasin Gishu and Nandi Counties of Kenya. 38 participants (19 M, 19 F) were adolescents between the ages of 13 and 17 from three schools. 19 students (10 male, 9 female) aged 13–17 attended a school in a rural part of the Nandi South District where almost all the students are primarily barefoot and very physically active [28]. The school is not directly accessible by road, and these students walk or run barefoot an average of 7.5 ± 3.0 km/day to travel to and from school [29]. A few of these students wear shoes a few hours a week when they attend church and other special occasions, but they are otherwise almost always barefoot (see below). We also recruited 9 female students aged 13–17 from a girl’s secondary school in Kobujoi, Kenya, and 10 male students aged 14–16 from a boy’s secondary school in Eldoret, Kenya. These students board at school and wear thick-soled leather shoes for most of the day, and either rubber sports shoes (plimsoles) or cushioned athletic shoes (trainers) during athletic activities. Finally, we recruited 10 habitually barefoot, male adults aged 23–60 from the Nandi South District, Kenya. These men walk long distances regularly, some still run several kilometers per week, and most of them ran long distances when they were younger.

**Table 1:** Levene's Test of unequal variance for nominal comparisons of foot strike angle (FSA).

Comparison	F-ratio	p-value
Sex (male vs female)	0.1349	0.7151
Footwear (bare vs shod)	2.5124	0.1197
Surface (hard vs soft)	6.1117	**0.0152**
Habitually barefoot	0.1062	0.7458
Habitually shod	0.081	0.7775

Individuals who had current lower extremity injuries or evident illness were excluded. In order to avoid biased samples in terms of fitness, we asked the teachers at the three schools to select only students who were “average” in terms of sports ability, thus excluding participants who either exceptional or poor in athletics.

### Ethics Statement

Approval for the human experimental study described in this paper was granted by the Harvard University Committee on the Use of Human Subjects (protocol F23121), and by the Moi University Medical Institutional Research And Ethics Committee (protocol 00695). As approved by the aforementioned committees, written informed consent for minors was provided by their teachers; informed consent was provided orally by adults who were unable to read and documented with their signature.

### Anthropometrics and Background Information

Basic anthropometrics were collected from all participants including height, body mass, and leg length (from the greater trochanter to the base of the heel). An orthopedic doctor (POM) examined all participants for lower extremity injuries. All participants (some of whom were not literate) were asked how far they walk and run on average each day, their regular physical activities, and what kinds of footwear they use. All questions were asked on two different occasions, either in Kalenjin or Kiswahili; one of the questioners (MS) speaks Kalenjin, knows the region intimately, and was able to evaluate how far each participant had to walk or run every day. Answers were then averaged. Since footwear usage and running history could not be quantified precisely as continuous variables, answers to these questions were binned into four rank order categories. Footwear score categories were: 1, almost always shod (less than 10% outdoor activity spent barefoot or in minimal shoes); 2, usually shod (mostly wear shoes, but do sports either barefoot or in minimal shoes); 3, mixed (sometimes walk, run or do physical activity in normal shoes and sometimes barefoot or in minimal shoes); 4, mostly barefoot (more than 80% of walking, running and physical activity done either barefoot or in minimal shoes). Running history categories were: 1, little (run less than 5 km/week); 2, occasional (run 5–10 km a week on an occasional but non-regular basis; 3, moderate (run 5–10 km a week on a regular basis); high (run >10 km a week on a regular basis).

### Experimental Trials

Participants were asked to wear whatever footwear they normally use (if applicable), and to wear shorts or skirts that could be rolled above the knee. In order to record 2-dimensional kinematics in lateral view, reflective tape markers were placed on the following locations on one side of the body: the greater trochanter, the center of the knee (in between the lateral femoral epicondyle and the lateral tibial plateau), the lateral malleolus, the lateral surface of the 5^th^ metatarsal head, and the lateral tuber calcaneus. Participants were then photographed with a visual scale in lateral and frontal position with a numeric identification. All participants were then instructed to run around an open field at a “pace they would choose if running a long distance” for approximately 5 minutes, at which point step frequency was then measured using an adjustable metronome (Matrix, New Market, VA, USA) fitted with an earpiece. Preferred step frequency (PSF) was recorded only for step frequencies that did not deviate by more than 4 steps/minute over a minimum of 30 seconds.

After warm-up, each participant’s kinematics was immediately recorded in lateral view on two adjacent tracks approximately 13–15 m in length. The “hard” track was the unaltered, grass-free, compact surface of a field, similar to the stiffness of a dirt road’s surface, and typical of the surfaces on which the participants normally run when traveling or doing athletics. A “soft” track was excavated parallel to the hard track by digging down 10 cm with a pickaxe, tamping down the earth, and then raking the dirt to create a smooth, soft surface. Penetrometer measurements repeated on each track (AMS Corp, American Falls, ID) indicate that the average compression strength of the hard track (3.85 kg/cm^2^±0.29 S.D.) was 5.5 times greater than the soft track (0.70 kg/cm^2^±0.27 S.D.). The soft track was raked between each set of trials, and re-excavated regularly to maintain a similar compliant surface for all participants. Small flags were used to mark the borders of the two tracks. A high-speed video camera (Casio EX-ZR100) was positioned at 0.7 m height approximately 4 m lateral to the 10 m point on the track, providing an additional 3–5 m of track beyond the field of the camera. All sequences were recorded at 240 frames per second.

For each trial, participants were asked to run down the track while looking forward and without decelerating until they had passed a marker positioned approximately 3 meters beyond the camera’s field of view. Participants were asked to run down both the hard and soft tracks at approximately 3.0 m/sec (“slow”) and 4.0 m/sec (“fast”) at several step frequencies: the previously determined preferred step frequency (PSF), and at 150, 170 and 190 steps/min. As a result, each participant ran a minimum of 16 conditions. Step frequency was controlled using a small, lightweight digital metronome either handheld or clipped onto clothing (Seiko DM50, SeikoUSA, Mahwah, NJ). For each trial, the participant was familiarized with the frequency and then asked to try to maintain that frequency for the entire length of the track. There was no landing target on the track in order to avoid having participants alter their gaits by either shortening or lengthening their steps as they passed the camera’s field of view. If the marked foot did not land in front of the camera, the trial was repeated without explaining the reason for repetition until a minimum of two trials were recorded for each speed, step frequency, and track. Following these trials, we administered the mile run test to the adolescent participants from each school according to methods outlined by the FITNESSGRAM test [30]. To avoid influencing how participants ran, we asked no questions about running form before or after the trials, and neither the participants nor their teachers were informed of the experiment’s objectives.

### Kinematic Analysis

All video sequences were converted to stacks of TIFF files and analyzed using ImageJ, version 1.46r (http://imagej.nih.gov/ij). A visual scale was determined for each participant using the measured distance between the lateral malleolus and knee markers. Since running speed was not controlled precisely during the experiment, running speed for each trial was quantified by measuring the horizontal translation of the marker on the greater trochanter between two homologous points during a stride cycle (e.g., toe-off to toe-off, or foot strike to foot strike for the same foot) relative to time (calculated from the number of frames divided by frame rate).

Foot strike was measured using only high-speed sequences in which the marked foot landed in front of the camera permitting a clear view of the foot’s lateral margin, which has been shown to yield high accuracy and reliability [31]. Foot strike angle (FSA) was quantified as a continuous variable by measuring the orientation of the calcaneus and 5^th^ metatarsal head markers relative to horizontal at the first frame of contact minus the same angle measured at foot flat [7]. Since FSA is a continuous variable but foot strike itself is a nominal variable, strike types were also classified using the following criteria: FFS, angles above 0.3°; MFS, angles between 0.3° and -5.6°; RFS, angles below -5.6°. In order to avoid classifying RFS and FFS landings as MFS landings, these cutoff values are more conservative than those used by Altman and Davis [31]. The correlation between strike type and FSA was 0.95 (p<0.0001).

Step frequency was quantified as the number of frames between the foot strike used to measure strike type and the previous strike multiplied by the number of seconds sampled per frame times 60. In order to quantify variations in the position of the foot at landing caused by variations in stride length, two measures of foot position relative to the rest of the lower extremity (overstride): overstride relative to the knee was measured as the projected anteroposterior distance of the lateral malleolus relative to the center of the knee at foot strike; overstride relative to the hip was measured as the projected anteroposterior distance of the lateral malleolus relative to the greater trochanter at foot strike. Several sagittal plane angles were measured at the moment of foot strike and at midstance (determined as the temporal midpoint between foot strike and toe-off). Knee angle was measured as the angle between the lines from the knee to the greater trochanter and the knee to the lateral malleolus; ankle angle was measured as the angle between the lines from the knee and to the lateral malleolus and from the lateral malleolus to the lateral MTP joint. Since this angle is affected by heel height, ankle angle was corrected by the angle measured during standing. Although hip angle is often measured as the orientation of the line from the knee to the greater trochanter relative to trunk angle, hip angle was measured as the orientation of the line from the knee to the greater trochanter relative to earth horizontal thus avoiding the effects of variations in trunk angle; similarly, trunk angle was measured as the angle between the greater trochanter and the center of the neck relative to earth horizontal.

Because each participant ran different numbers of trials, all kinematic measurements were averaged for each individual and condition. Measurement reliability was quantified by taking the same set of angular measurements from one individual on five separate occasions [11]. The average standard deviation was 0.32° with a range of 0.18°-0.49°. In addition, a test-retest sensitivity analysis conducted by taking all measurements twice from the same trial, yielding a correlation coefficient of 0.927.

### Statistical analyses

As described above, this study tests hypotheses about levels of variation in foot strike (H1) as well as the factors that influence this variation (H2). In order to test the effects of nominal and continuous variables on overall levels of variation, as predicted by H1, we used two different methods. First, we used Levene’s Test to compare measured variance of the two foot strike variables, FSA and strike type, in relation to the three nominal variables studied: sex, footwear condition (barefoot versus shod), and surface stiffness (hard versus soft). To test if footwear condition affects foot strike variability on the two surfaces, Levene’s Test was also used to compare foot strike variance on the hard versus soft trackways within the barefoot and shod participants. To account for repeated measures, these tests used the mean variance of each individual. A Chi-squared analysis was also used to test if the proportion of individuals who varied their strike type differed between the barefoot and shod participants. Second, to test if there is a relationship between levels of variation in foot strike and intrinsic, extrinsic and acquired factors that are continuously distributed, we used a bivariate General Linear Mixed Model (GLMM) to calculate the residuals of the regression between FSA and each predictor variable using a subject identifier as the random effect to account for the non-independent error generated by repeated measures on the same individuals [32]. We then used a second GLMM to regress the absolute value of these residuals against the relevant predictor variable. A slope (the coefficient of the GLMM) significantly different from zero indicates a significant increase or decrease in variation with respect to the predictor variable. Since GLMMs assume that variables are normally distributed and in comparable units, non-normally distributed variables (assessed using a Shapiro-Wilk test) were log-transformed, and then all variables were converted to Z-scores.

In order to test Hypothesis 2, we used multivariate GLMMs to model the effects of the intrinsic, extrinsic, and acquired variables on foot strike across treatments. In the first GLMM, the dependent variable was FSA was regressed against the fixed effects included several variables classified as extrinsic (substrate stiffness), intrinsic (age, sex, height, body mass), and acquired factors (footwear history and running history; preferred step frequency; and the speed at which the participants could run a mile, a proxy for overall physical fitness). The first GLMM took the following form:

$$\begin{array}{l} \text{FSA}=\beta_{1}\text{Surface}+\beta_{2}\text{Age}+\beta_{3}\text{Sex}+\beta_{4}\text{Height}+\beta_{5}\text{Body Mass}+\beta_{6}\text{Footwear History}+\\ \beta_{7}\text{Running History}+\beta_{8}\text{Preferred Step Frequency}+\beta_{9}\text{Mile Time}+ZU+Є \end{array}$$

A second GLMM (S1 Table) was also calculated to test the effects of kinematics on foot strike. In this GLMM, the dependent variable was FSA and the fixed effects were aspects of kinematics (speed, step frequency, trunk angle, hip angle, knee angle, and overstride relative to the knee). This took the following form:

$$\begin{array}{l} \text{FSA}=\beta_{1}\text{Speed}+\beta_{2}\text{Step Frequency}+\beta_{3}\text{Trunk Angle}+\beta_{4}\text{Hip Angle}+\beta_{5}\text{Knee Angle}+\\ \;\beta_{6}\text{Ankle Angle}+\beta_{7}\text{Overstride}+ZU+Є \end{array}$$

In both models, β_i_ is the fixed-effect coefficient for the *i*th predictor, *Z* is the design matrix for the random grouping variable, *U* is a vector of random effects, and Є is residual model error. A subject identifier was used as the random grouping effect to account for repeated measures on the same individuals. In addition, all variables were transformed to Z-scores, and non-normally distributed variables (assessed using a Shapiro-Wilk test) were log-transformed.

A few of the variables (including the primary outcome measurement, FSA) were not normally distributed after transformation to Z-scores, and some variables are highly collinear (e.g., height and body mass, speed and step frequency). Therefore, to account for non-normality and isolate the potential effects of multicollinearity on significance testing, we also used a non-parametric residual randomization method to calculate p-values in the GLMMs. Residual randomization is a type of permutation test in which statistical significance is tested by permuting the residuals of a model rather than the observations [33]. In this study, residual randomization is used to evaluate the significance of each variable’s effect independently, removing partial effects of other collinear variables (see S1 File).

## Results

Because this study aimed to sample a wide range of variation both within and between different groups, we begin with a summary of the variation sampled. In terms of external factors, average height was 160.9 ±9.5 cm (range 142–177) in the barefoot population and 162.3±8.1 cm (range 147–174) in the shod population (p = 0.517); average body mass was 47.0±8.4 kg (range 32–62) in the barefoot population and 54.0±8.1 kg (range 37–65) in the shod population (p = 0.038); average age was 24.8±15.1 years (range 13–37) in the barefoot population, and 15.4 ±1.07 years (range 13–18) in the habitually shod population. In terms of acquired variables measured, the average footwear score in the barefoot population was 3.34±1.2 (range 2–4), higher (p<0.001) than the average shod population score of 1.20±0.4 (range 1–2); the average running history score in the barefoot population was 2.86±1.3 (range 1–4), also higher (p<0.001) than the average shod population score of 1.37±0.6 (range 1–3); however, in both populations there were individuals who ran more than 10 km per week and those who ran infrequently (<5 km/week). The average mile time of the barefoot individuals was 1:38 faster than the shod individuals (p = 0.003), with ranges of 5:19–7:42 and 5:29–12:20, respectively. Preferred step frequency averaged 172.6±7.7 steps/minute (range 152–185) in the barefoot population, higher (p<0.001) than the average in the shod population of 159.2±8.0 steps/minute (range 150–172).

**Fig 1:**
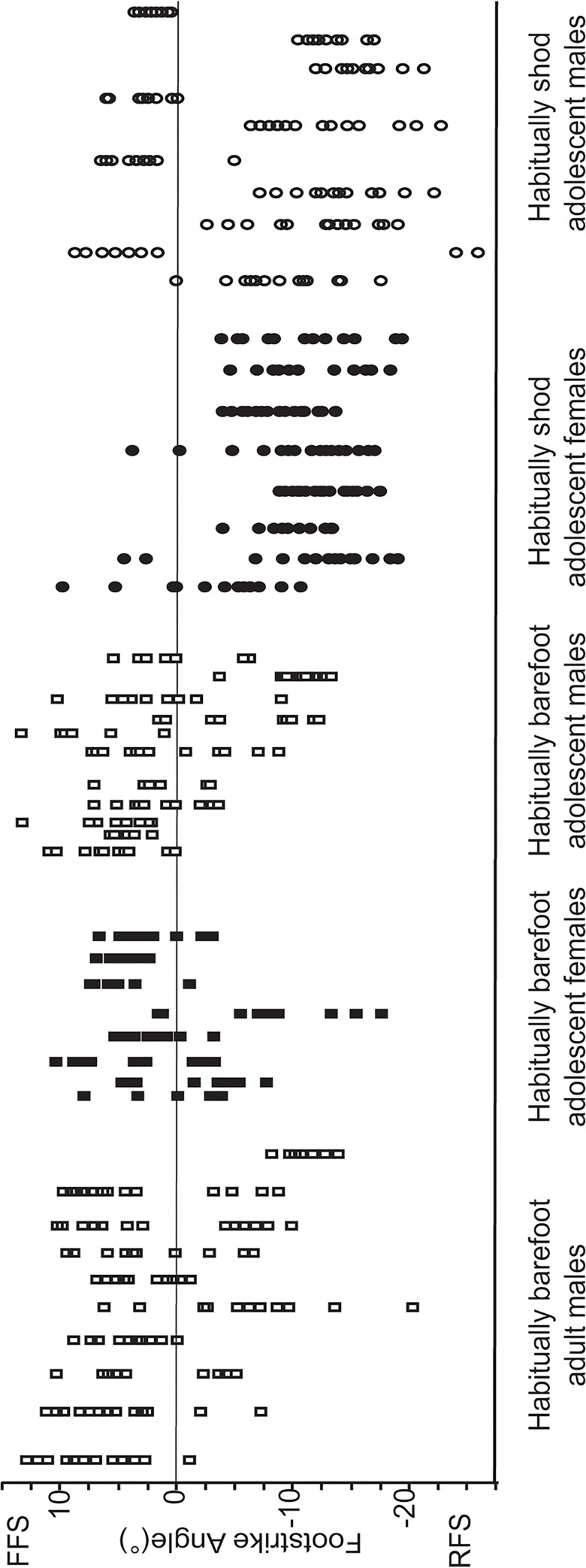
Variation in foot strike angle (FSA). Every FSA measured for every participant, noting which are forefoot (FFS), midfoot (MFS) and rearfoot (RFS) strikes. Note the greater degree of variability in the habitually barefoot individuals.

Fig 1, which graphs the FSA and strike type of every trial of every subject, highlights the considerable variation in foot strike observed within subjects as well as between groups. Although average FSA was 1.1° ±5.3 among the habitually barefoot individuals and -8.3° ±6.1 among the habitually shod individuals, indicating that average strike type for each group was a FFS and RFS, respectively, a slight majority of individuals (56%) used more than one strike type. The average intra-individual variance for FSA was 20.65° ±3.12 s.e.

The first hypothesis, H1, tested predictions of higher levels of variation in foot strike with respect to several nominal variables including sex, footwear use (barefoot versus shod) and surface stiffness (hard versus soft trackways), as well as in continuous variables such as speed, running history, and footwear history. Levene’s Tests of nominal comparisons for FSA, summarized in Table 1, indicate that variation in FSA was not greater in men than women, or among individuals who were barefoot than shod. However, a Chi-square test revealed that the percentage of barefoot individuals who used more than one strike type (72%) was significantly greater than the percentage of shod individuals who used varied strike types (32%) (Pearson χ^2^ = 7.78 (1); p = 0.005). Although the entire study sample had significantly more foot strike variation when running on soft than on hard surfaces (p<0.05), this difference was not significant within the barefoot population or within the shod population.

Since most of the variables analyzed in this study are continuous, GLMMs were used to calculate the residuals of mixed-effect regressions between FSA and each predictor variable (Table 2). Of the continuously distributed extrinsic variables studied, speed had no significant effect on variation in FSA, but individuals used more variable foot strikes (p = 0.05) when they ran with lower step frequencies. Finally, of the acquired factors measured, variation in FSA was homogenous for preferred step frequency, footwear history or mile time on the degree of foot strike variation, but individuals who ran more frequently had considerably less variation in foot strike variation (p<0.0001) than individuals who ran less.

**Table 2:** GLMM analysis of variation in foot strike angle (FSA) relative to continuously distributed predictor variables.

Variable	Coefficient	S.E.	t-value	p-value
*Intrinsic factors*				
Age	-0.025	0.419	-0.83	0.41
Weight	0.000	0.119	0.00	1.00
Height	-0.019	0.413	0.47	0.65
*Extrinsic factors*				
Speed	0.015	0.021	0.72	0.47
Step freq	0.052	0.026	1.97	**0.05**
*Acquired factors*				
PSF	-0.007	0.026	-0.28	0.78
Footwear history	-0.013	0.026	-0.50	0.62
Running history	-0.160	0.039	-0.42	**<0.001**
Mile time	0.045	0.059	0.76	0.45

The second hypothesis, H2, focused on what extrinsic, intrinsic and acquired factors influence FSA in the population studied. We predict that FSA would be significantly affected by speed, step frequency, and surface stiffness, as well as footwear and running history. The hypothesis was tested using a GLMM, summarized in Table 3, with FSA as the response variable, and in which the fixed effects included age, sex, height, body mass, substrate stiffness, footwear history, running history, preferred step frequency, and the speed at which the participants could run a mile. As the coefficients (which represent the slope of the relationship between FSA and each predictor variable) in Table 3 indicate, none of the intrinsic variables (sex, age, body mass) have an effect on FSA at conventional levels of significance (p<0.05), but there were marked, significant effects on FSA (in order of t-value) from preferred step frequency (p = 0.001), footwear history (p = 0.011) and track surface (p = 0.02); running history (p = 0.086) and mile time (p = 0.083) trended toward conventional levels of significance. In other words, individuals were more likely to have a higher FSA and thus FFS or MFS independent of speed and anthropometric characteristics if they used a higher step frequency, rarely used shoes, and ran on a soft surface; and they had a greater tendency to FFS or MFS if they had more experience running and had faster mile times.

**Table 3:** GLMM analysis of effects of intrinsic, extrinsic and acquired variables on foot strike angle (FSA)*.

Variable	Coefficient Estimate	Std. Error	t-value	Standard parametric p-value	Residual Randomization p-values
Surface	0.1224	0.0474	2.585	**0.0101**	**0.015**
Age	0.5341	0.6462	0.8265	0.4167	0.303
Sex	0.401	0.2931	1.3681	0.184	**0.089**
Height	0.0211	0.1568	0.1348	0.8939	0.791
Body mass	-0.1226	0.1857	-0.6602	0.5154	0.304
Footwear History					**0.011**
Footwear 2	-1.0245	0.4338	-2.3615	**0.0267**	
Footwear 3	-1.5676	0.6289	-2.4928	**0.02**	
Footwear 4	-0.9965	0.6289	-1.5847	0.1261	
Running History					**0.086**
Running 2	0.1368	0.3383	0.4043	0.6895	
Running 3	1.2861	0.57	2.2562	**0.0334**	
Running 4	1.412	0.5843	2.4167	**0.0236**	
Preferred Step Frequency	0.6667	0.2063	3.2326	**0.0035**	**0.001**
Mile Time	0.2808	0.2296	1.2226	0.2333	**0.083**

*Fixed effects multiple R-squared: 0.76, Fixed effects adjusted R-squared: 0.75.

**Fig 2:**
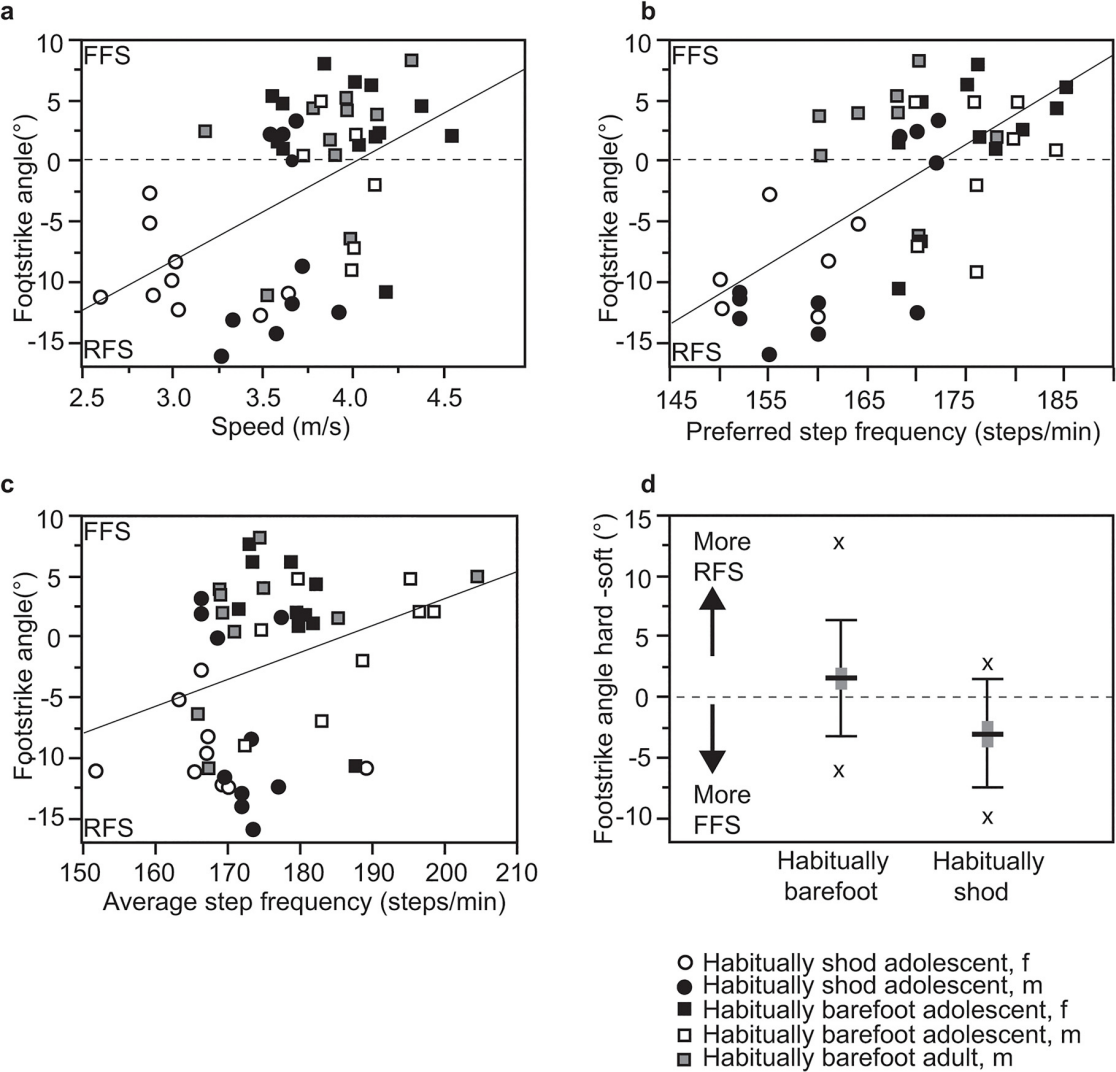
Sources of variation in foot strike angle (FSA). a) Regression of speed versus FSA; b) regression of measured step frequency versus FSA; c) regression of preferred stride frequency versus FSA; d) Box (standard error) and whisker (standard deviation) plot of difference in FSA on hard versus soft tracks for habitually barefoot and shod individuals (more positive values indicate more dorsiflexed FSA on soft surface; more negative values indicate more plantar flexed FSA on soft surface); x marks indicate maximum and minimum values.

**Fig 3:**
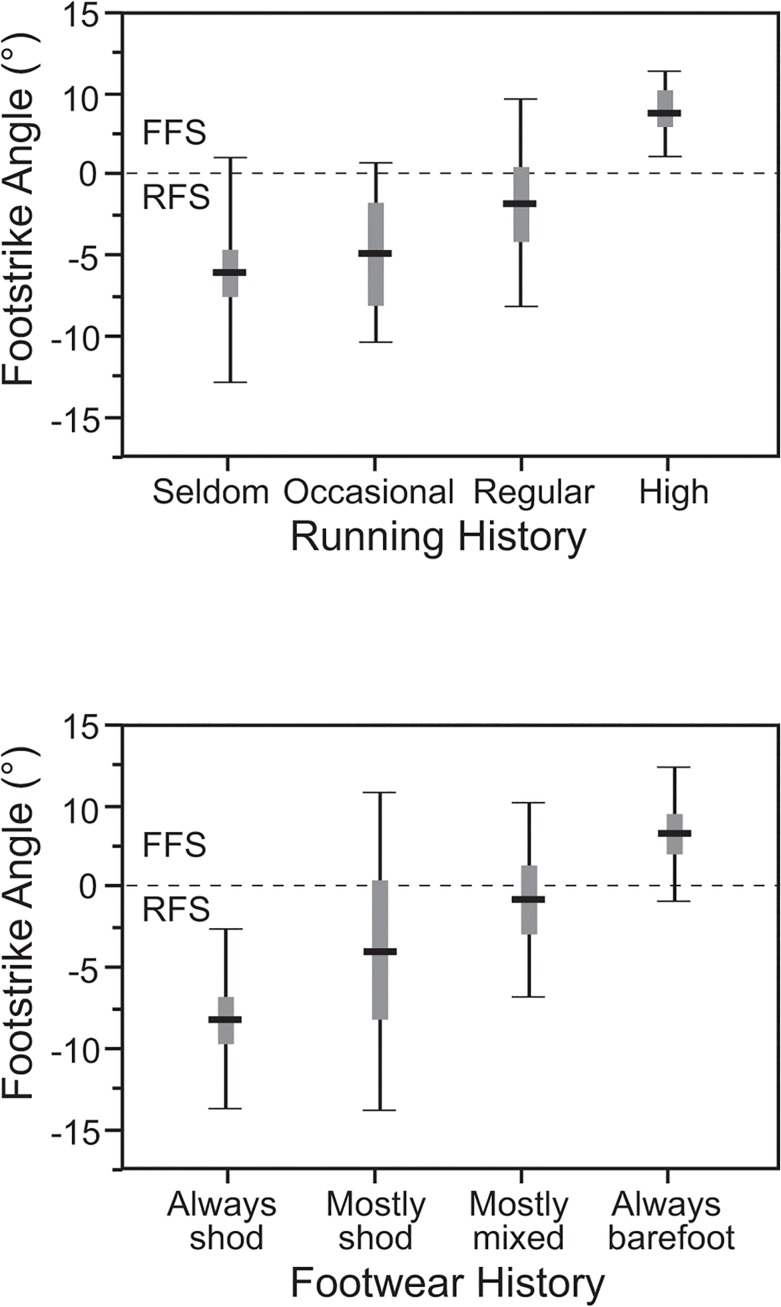
Foot strike angle (FSA) and running history and footwear history. Box (standard error) and whisker (standard deviation) plots of average FSA (°) for individuals categorized by running history (a) and by footwear history (b). See text for explanation of how participants were binned into categories. In both analyses, p<0.001 (oneway ANOVA).

Figs 2 and 3 further explore the conventional bivariate associations between averaged FSA and selected intrinsic, extrinsic and acquired variables. In terms of speed, the habitually barefoot participants ran about 18% faster than the habitually shod participants, leading to a significant correlation (r = 0.49; p = 0.004) between speed and FSA within the population as a whole, but not within the habitually barefoot (r = 0.04; p = 0.83) and habitually shod (r = 0.16; p = 0.51) groups (Fig 2A). Measured step frequency was uncorrelated with FSA either within or between groups (Fig 2B), but preferred step frequency correlated strongly with FSA in the population as a whole (r = 0.692; p<0.001) and within the habitually shod runners (r = 0.652; p = 0.002), and approached conventional levels of significance within the habitually barefoot groups (r = 0.333; p = 0.07) (Fig 2C). To assess the effects of surface stiffness on foot strike, Fig 2D graphs the difference in average FSA on the hard versus soft tracks, with a value of zero indicating no difference, and positive or negative values indicating a greater tendency to RFS or FFS on soft surfaces, respectively. As this analysis shows, habitually barefoot individuals were more likely to RFS on the soft track with average FSAs that were 1.88°± 0.85 (s.e.) more dorsiflexed (t-test = 1.71, p = 0.04); in contrast, habitually shod individuals were more likely to FFS with average FSAs that were 2.16°± 0.95 (s.e.) more plantar flexed (t-test = -5.83, p<0.001).

As noted above, it was not possible to quantify running history and footwear history as continuous variables, but Fig 3 summarizes the relationship between binned categories of these acquired variables and FSA. As Fig 3A shows, individuals who spend more time barefoot show considerable variation in FSA but tend to have more positive FSAs, whereas individuals who are more habitually shod have more negative FSAs (ANOVA, p<0.001). Similarly, individuals who run more have significantly higher FSAs, reflecting a higher percentage of FFS (Fig 3B). Because running and footwear history are not independent in this population, we tested the effects of multicollinearity using partial correlation analysis. The partial correlation of running history with FSA holding constant the effects of footwear history is 0.31 (p = 0.04), and the partial correlation of footwear history with FSA holding constant running history is 0.45 (p = 0.001), indicating that both of these acquired factors contribute independently to foot strike variation.

Finally, since the focus of this study was on variation in FSA, a second GLMM was computed to explore the effects of running kinematics on FSA. The results of this analysis (S1 Table), indicate that FSA was most influenced by ankle angle, overstride relative to the knee, and trunk angle (p = 0.001), and was not strongly associated with speed and hip angle. An ANOVA, however, revealed some significant differences in kinematics between the habitually barefoot and shod individuals. In particular, the habitually barefoot individuals had 8% higher average preferred step frequencies (172 vs 159 steps/min, p<0.0001); tended to land with 5–6° more flexed knees and hips (p<0.001); had approximately 50% less overstride relative to the knee (p = 0.0007); and had 4° more vertical trunks (p = 0.002). Some of these differences may be attributable to speed, which was 0.6 m/s higher in the habitually barefoot individuals, largely because the habitually unshod adolescent males ran approximately 0.5 m•s^-1^ faster (4.04 m•s^-1^ ± 0.31) than the population mean of 3.69 m•s^-1^ ± 0.44, and the habitually shod adolescent females ran significantly slower (3.04 m•s^-1^ ± 0.32) (ANOVA, p<0.0001). Group means for these kinematic variables are summarized in S2 Table.

## Discussion

The most basic result of this study is that under varied running conditions foot strike angle and type can be variable both within and between individuals, especially among habitually barefoot individuals. This variation is highlighted by the plot of every FSA recorded in the study (Fig 1), which shows that the average intra-individual standard deviation of FSA was 4.12°, and that while a majority of participants (56%) used a combination of FFS, MFS and RFS landings, 72% of the barefoot runners and 32% of the shod runners used multiple strike types.

This study tested two general hypotheses regarding the effects of intrinsic, extrinsic, and acquired factors on variations in observed in foot strike. The first general hypothesis—that certain factors influence the degree of variation in strike type—was supported (see Table 1 and Table 2). Although none of the intrinsic factors measured (height, sex, age, and body mass) affected the degree of variation in FSA, several extrinsic and acquired factors did influence FSA variation. In particular, there was a significantly greater degree of FSA variability within individuals who used lower step frequencies and who typically ran less. In addition, although FSA variation was not affected by footwear history, individuals who were barefoot had significantly more variable foot strike types than those who were wearing shoes. The explanation for this seemingly contradictory result is that average FSA among barefoot individuals was 1.11° ± 5.3, whereas the average FSA among those who were habitually shod was -8.3° ± 6.1 (t-test, p<0.001). Consequently, individuals who were barefoot were more likely to not only FFS and MFS, but also to sometimes land with negative values (a RFS), while habitually shod individuals were less likely to land with flat or plantar flexed feet. Note also that there was no effect of preferred step frequency, mile time, surface stiffness, or speed on the degree of FSA variation.

The second general hypothesis tested was that a combination of intrinsic, extrinsic, and acquired factors would influence FSA, hence strike type. In particular, it was predicted that participants would be more likely to shift to more positive FSA values, hence a higher frequency of MFS or FFS landings, when they ran at faster speeds, higher step frequencies and on harder surfaces, and that participants who were more experienced runners or were habitually barefoot would also be more likely to MFS or FSS. These hypotheses were all supported. In particular, the GLMM (Table 3) revealed significant effects of track stiffness, preferred step frequency, footwear history, running history and mile time speeds. Put simply, individuals were less likely to RFS when they ran on a harder track (Fig 2D), preferred higher step frequencies (Fig 2C), were able to run faster, were experienced runners (Fig 3A), and were habitually barefoot (Fig 3B). In contrast, there was no effect on FSA from age, sex, body mass, height, or speed (Fig 2A).

These results are consistent with a previous, smaller comparison of barefoot and shod Kalenjin individuals that sampled a more limited range of faster speeds [7], as well as studies that compare running form among populations that vary in footwear use [11,15] or in which habitually shod individuals have been studied both barefoot and shod [8,9,34]. Although barefoot individuals sometimes RFS, they are more likely to FFS and MFS depending on conditions and experience; in contrast, habitually shod individuals are more likely to RFS under a range of conditions.

Although not a focus of this study, the results presented here confirm those of previous studies that compared kinematics and kinetics between barefoot and shod runners [4–12,15]. In general, the habitually barefoot participants landed with more flexed knees and hips, they had slightly more vertical trunks, they preferred higher step frequencies, and they were less likely to overstride (S2 Table). When a GLMM was used to tease apart which of these variables were associated with variations in FSA, the strongest predictor was ankle angle, with significant associations also evident for overstride and trunk angle (S1 Table).

Before considering the implications of these results, it is worth summarizing the study’s limitations. One problem is the limited range of subjects, conditions, and factors sampled. We were unable to include adult women, and the sample sizes for each group were necessarily limited by time and opportunity. Broadening the sample in terms of age, sex, running experience, and footwear history would likely reveal additional variability. In addition, the experimental design did not look at fatigue, which can increase the likelihood of using a RFS [35], and only a few external factors hypothesized to influence kinematics (notably speed, step frequency and surface stiffness) were manipulated. Future studies would benefit from examining substrate factors such as slipperiness, smoothness, inclines, and changes in direction of the sort that runners encounter when they run on trails and other variable environments that, until relatively recently, were the primary contexts in which people ran. Another necessary limitation of the study was to measure only sagittal plane kinematics using video without collecting information on ground reaction forces and muscle function. A final concern was the participants’ ability to run normally. Although the experiment was not conducted in the laboratory on a treadmill, running at different speeds on a track with markers taped to one’s joints while trying to adapt one’s step frequency to a metronome is an unusual experience that can interfere with normal running form. This concern, however, applies to all studies of running kinematics and kinetics, and it is arguable that the conditions tested here are a step in the direction of understanding variability in running form beyond the laboratory and among individuals who are not just habitually shod from developed countries. Although such people are the focus of most research, they are unusual from an evolutionary perspective [36].

These limitations aside, the study’s results have some relevance for current discussions about running form. Most importantly, very few studies on running biomechanics have sampled runners who are not habitually shod and in the natural settings in which people used to locomote rather than in controlled laboratory conditions, primarily on treadmills or over force plates [7,11,13,14]. It is reasonable to hypothesize that these modern contexts limit variation in foot strike as well as other aspects of kinematics. The results presented here raise the possibility that for much of human evolution foot strike patterns were more variable. The two most obvious factors that have potentially contributed to less variation in how people run is increased use of flat, paved surfaces and treadmills for running, and the prevalence of running shoes with elevated, cushioned heels that have been available only since the 1970s [7]. Just as cushioned heels facilitate RFS landings on hard surfaces, it is reasonable to hypothesize that the barefoot individuals measured in this study were more likely to run with a RFS on the soft trackway because softer substrates, like cushioned heels, make RFS landings more comfortable by lowering the rate of loading of the impact peak [7]. Although soft and smooth surfaces no doubt existed in the past such as along lakeshores and in sandy environments, most people typically walked and ran on compacted soil with rocks, vegetation, and features that increase substrate complexity and stiffness. Walking and running without shoes on these surfaces unquestionably elicits much more varied and extreme stimuli from sensory nerves on the glabrous surface of the sole. It is therefore reasonable to hypothesize that people ran with more varied kinematics prior to the invention of shoes, which probably occurred in the last 40,000 years [37].

Another factor that may have affected variation in running form is skill. Since the running boom that began in the 1970s, there has been an increase in running among amateurs, who usually get less coaching and train less intensively than athletes who are professional or on teams [38]. One hypothesis that merits further testing is that untrained, amateur runners in developed nations are more likely to run like the habitually shod Kalenjin studied here, with a relatively slower step frequency and a greater proclivity to land with a dorsiflexed foot, hence a RFS. This observation leads to the hypothesis that a contributing factor to Kalenjin excellence in distance running might be that most elite Kalenjin runners grew up running long distances without shoes on a regular basis in the same conditions as the habitual barefoot participants analyzed in this study [27]. Although habitually barefoot people from the Daasanach tribe in northern Kenya were observed to mostly run with a RFS at slow speeds (2.1–3.0 m/s) a possible explanation for this different result, apart from speed, is that these individuals live in a hot, sandy desert and do not run often much [14]. Other studies of adults from habitually barefoot and minimally shod populations found that individuals (especially men) were more likely to FFS or MFS [7,11,13].

Finally, what do these results mean for habitually shod individuals who run mostly on pavement and treadmills, and wonder how to make sense of diverse arguments about minimal shoes, cushioning, and strike type? First, the restricted variation in strike type among habitually shod runners today may be a recent phenomenon, and it would be useful to test if runners adopt more variation when running on trails rather than on pavement or treadmills. In addition, although rearfoot and forefoot striking are both normal, everything involves trade-offs. RFS landings have the potential advantages of being comfortable in shoes or on soft surfaces, they require less calf and foot muscle strength, and they lessen external moments acting around the ankle [39]. Their potential disadvantages are that they cause impact peaks whose rate and magnitude are hypothesized by some researchers to be related to some repetitive stress injuries, they increase external moments around the knee, and certain kinematic patterns associated with (but not exclusive to) RFS gaits, such as overstriding and extended knees at landing, are implicated in some repetitive stress injuries [40]. More research is needed to evaluate the costs and benefits of different strike types, but one hypothesis that also needs to be explored is that running with more kinematic variation, as perhaps occurs during trail running, is more natural and may also be beneficial.

## Supporting Information

S1 File Residual Randomization Methods. (DOCX)

S1 Table GLMM analysis of effects of kinematic variables on Foot strike Angle (FSA)*. (DOCX)

S2 Table Group means and standard deviations for major variables studied. (DOCX)
