# Supplementary material for: Variation in Foot Strike Patterns among Habitually Barefoot and Shod Runners in Kenya
Source: PLoS One. 2015 Jul 8;10(7):e0131354. doi: 10.1371/journal.pone.0131354 (PMC4495985; doi:10.1371/journal.pone.0131354)
Supplement: S1 File — (DOCX) [file pone.0131354.s001.docx]

**Supplemental Material 2 Text: Residual Randomization Method**

As noted previously, to account for non-normality and isolate the potential effects of multicollinearity on significance testing, we also used a non-parametric residual randomization method to calculate p-values in the GLMMs. In the case of multiple regression, when one wishes to test the effect of each predictor on the response (while accounting for the covariance among predictors), the procedure detailed by Anderson [1] is recommended. Rigorous investigation of this test has revealed desirable type I and type II error rates in the context of regression [1,2].

To demonstrate the residual randomization methodology, consider the following simplified regression model:

Y ~ X_1_ + X_2_

where the dependent variable, Y, is regressed against two independent variables, X_1_ and X_2._ In this scenario, variables Y, X_1_, and X_2_ do not meet assumptions of normality. To test individually for X_1_as a main effect, while maintaining the covariance structure between Y, X_1_, and X_2_, the partial effect of X_1_ on Y is removed by regressing Y ~ X_1_. We then obtain regression coefficients B and compute the residuals R, as R = Y – X_1_B. We can then regress, R ~ X_1_ + X_2_, measuring the individual effect of X_2_ on Y while maintaining its covariance of X_2_ with X_1_. We can extend this same procedure to test the main effects of multiple independent X predictor variables while maintaining their covariance with Y.

As a device test statistic, we know that the larger the F statistic, the stronger the effect of some variable on the response. Thus, to test the effect of X_1_ or X_2_ on Y we compute the observed F statistic. We then permute (shuffle) the partial residuals, obtain a permuted F value (F-perm) and compare it to the observed F value (F-obs). This is repeated 1000 times to generate a distribution of permuted F statistics. P-values are calculated by comparing the probability of obtaining a more extreme F-obs, computed by counting the number of F-perms that are greater than or equal to F-obs, then dividing them by the number of permutations. In our case, the residual randomization method obtains an F-statistic for every coefficient in the GLMM, and the null hypothesis is that the estimated coefficient effects can be obtained by random chance alone.

1. Anderson MJ (2011) Permutation Tests for univariate or multivariate analysis of variance and regression. *Can J Fisheries Aquatic Sci* 58:626-639.

2. Manly BFJ (2006)  Randomization, Bootstrap, and Monte Carlo Methods in Biology. 3rd ed. Boca Raton: CRC Press. 399 p.
