# Supplementary material for: Variation in Foot Strike Patterns among Habitually Barefoot and Shod Runners in Kenya
Source: PLoS One. 2015 Jul 8;10(7):e0131354. doi: 10.1371/journal.pone.0131354 (PMC4495985; doi:10.1371/journal.pone.0131354)
Supplement: S1 Table — (DOCX) [file pone.0131354.s002.docx]

| **S1 Table:** GLMM analysis of effects of kinematic variables on Foot strike Angle (FSA)* | | | | | |
| --- | --- | --- | --- | --- | --- |
| Variable | Coefficient Estimate | Std. Error | t-value | Standard parametric p-value | Residual randomization p-values |
| Speed | -0.029 | 0.028 | -1.043 | 0.298 | 0.308 |
| Step Frequency | 0.044 | 0.025 | 1.758 | **0.0795** | 0.111 |
| Trunk Angle | 0.140 | 0.025 | 5.663 | **<0.0001** | **0.001** |
| Hip Angle | 0.015 | 0.044 | 0.334 | 0.739 | 0.741 |
| Knee Angle | -0.067 | 0.044 | -1.539 | 0.124 | 0.117 |
| Ankle Angle | 0.535 | 0.030 | 17.820 | **<0.0001** | **0.001** |
| Overstride rel. to knee | -0.387 | 0.036 | -10.617 | **<0.0001** | **0.001** |
|  |  |  |  |  |  |
| *Fixed effects multiple R-squared: 0.59, Fixed effects adjusted R-squared: 0.58 | | | | | |
