# Supplementary material for: Variation in Foot Strike Patterns among Habitually Barefoot and Shod Runners in Kenya
Source: PLoS One. 2015 Jul 8;10(7):e0131354. doi: 10.1371/journal.pone.0131354 (PMC4495985; doi:10.1371/journal.pone.0131354)
Supplement: S2 Table — (DOCX) [file pone.0131354.s003.docx]

**S3 Table:** Group means and standard deviations for major variables studied

| Group | N | Sex | Avg. speed (m/sec) ±s.d. | Strike type mode | Strike type mean ±s.d. | FSA (°) ±s.d. | Ankle angle (°) ±s.d. | Knee angle (°) ±s.d. | Hip angle (°) ±s.d. | Trunk angle (°) ±s.d. | Preferred Step Freq. ± s.d. | Over-stride (m) ±s.d. |
| --- | --- | --- | --- | --- | --- | --- | --- | --- | --- | --- | --- | --- |
| Barefoot adolescent | 9 | F | 3.82±0.21 | F | 1.94±0.69 | 0.00±5.24 | 117.2±3.8 | 150.1±5.2 | 54.3±2.8 | 83.6±2.0 | 176.9±4.7 | 0.02±0.01 |
| Barefoot adolescent | 10 | M | 4.04±0.31 | F | 1.64±0.64 | 2.17±5.19 | 115.8±4.9 | 152.8±5.7 | 55.5±3.8 | 87.3±2.9 | 175.6±5.9 | 0.04±0.02 |
| Barefoot adult | 10 | M | 3.86±0.32 | F | 1.76±0.65 | 1.05±5.70 | 114.0±3.7 | 155.4±4.1 | 62.8±3.2 | 80.6±3.1 | 165.8±7.2 | 0.02±0.02 |
| Shod adolescent | 10 | F | 3.51±0.22 | R | 2.29±0.91 | -7.15±7.81 | 117.5±8.0 | 155.8±4.1 | 60.1±4.1 | 80.0±4.0 | 162.9±8.5 | 0.23±0.04 |
| Shod adolescent | 9 | M | 3.04±0.32 | R | 2.86±0.22 | -9.62±3.41 | 116.0±3.2 | 161.4±4.8 | 67.8±3.5 | 80.2±2.5 | 155.1±5.2 | 0.11±0.83 |
|  |  |  |  |  |  |  |  |  |  |  |  |  |
| Unshod combined | 30 | M+F | 3.93±0.29 | F | 1.77±0.65 | 1.11±5.30 | 115.6±4.2 | 152.9±5.3 | 57.6±5.0 | 83.85±0.7 | 172.6±7.7 | 0.03±0.02 |
| Shod combined | 19 | M+F | 3.33±0.38 | R | 2.56±0.72 | -8.3±6.11 | 116.8±6.1 | 158.4±5.2 | 63.7±5.5 | 80.11±0.9 | 159.2±8.0 | 0.05±0.03 |
| HS vs HB |  |  | p<0.0001 |  | p=0.0003 | p<0.0001 | p=0.479 | p=0.0009 | p=0.0002 | p=0.002 | p<0.0001 | p=0.0007 |
